# Exploiting the neuroprotective effects of α-klotho to tackle ageing- and neurodegeneration-related cognitive dysfunction

**DOI:** 10.1042/NS20200101

**Published:** 2021-06-14

**Authors:** Kelsey Hanson, Kate Fisher, Nigel M. Hooper

**Affiliations:** 1 Division of Neuroscience and Experimental Psychology, School of Biological Sciences, Faculty of Biology, Medicine and Health, University of Manchester, Manchester M13 9PT, U.K.; 2 Geoffrey Jefferson Brain Research Centre, Manchester Academic Health Science Centre, Northern Care Alliance and University of Manchester, Manchester, U.K.

**Keywords:** aging, Alzheimers disease, cognition, neurodegeneration, neuroprotection

## Abstract

Cognitive dysfunction is a key symptom of ageing and neurodegenerative disorders, such as Alzheimer’s disease (AD). Strategies to enhance cognition would impact the quality of life for a significant proportion of the ageing population. The α-klotho protein may protect against cognitive decline through multiple mechanisms: such as promoting optimal synaptic function via activation of N-methyl-d-aspartate (NMDA) receptor signalling; stimulating the antioxidant defence system; reducing inflammation; promoting autophagy and enhancing clearance of amyloid-β. However, the molecular and cellular pathways by which α-klotho mediates these neuroprotective functions have yet to be fully elucidated. Key questions remain unanswered: which form of α-klotho (transmembrane, soluble or secreted) mediates its cognitive enhancing properties; what is the neuronal receptor for α-klotho and which signalling pathways are activated by α-klotho in the brain to enhance cognition; how does peripherally administered α-klotho mediate neuroprotection; and what is the molecular basis for the beneficial effect of the VS variant of α-klotho? In this review, we summarise the recent research on neuronal α-klotho and discuss how the neuroprotective properties of α-klotho could be exploited to tackle age- and neurodegeneration-associated cognitive dysfunction.

## Introduction

Ageing is the primary risk factor for cognitive decline and most neurodegenerative disorders. Cognitive dysfunction is the major symptom of Alzheimer’s disease (AD), as well as being prominent in other forms of dementia. Thus, strategies to enhance cognition would impact on the quality of life for a significant proportion of the ageing population. *α-klotho* is a key anti-ageing gene: in mice its deficiency results in premature ageing and short lifespan [1], while its overexpression extends lifespan [2,3]. In humans, a genetic variant of *α-klotho* is associated with enhanced cognition [3]. In mouse models, α-klotho protected against both age-associated decline in cognitive performance and neurodegenerative disease-associated cognitive dysfunction (reviewed in [4]). These observations have led to α-klotho being considered as a potential neuroprotective and cognitive-enhancing agent. However, the molecular and cellular mechanisms underpinning these observations are far from complete.

The klotho (KL) family of genes includes *α-klotho*, *β-klotho* and *γ-klotho* [5], which are all translated as single-pass transmembrane proteins. α-klotho is highly expressed in the brain and kidney, and to a lesser extent in other organs [6]. In the periphery, transmembrane α-klotho acts as a co-receptor for FGF23 to increase binding affinity to fibroblast growth factor (FGF) receptors. β-klotho is predominantly expressed in the liver, with lower levels present in the gut, kidney and spleen and mediates the activity of other members of the FGF family, mainly FGF-19 and FGF-21 [7,8]. γ-klotho, whose function is ill-defined, is expressed in the kidney and skin [6,7,9]. In this review, we outline the molecular and cellular properties of α-klotho (referred to hereafter as klotho), its neuroprotective functions and the role of the VS variant in enhancing cognitive ability. In addition, we highlight critical gaps in our knowledge of the mechanisms by which klotho confers neuroprotection; gaps which if filled may open new therapeutic approaches to mimic klotho activity in age- and neurodegeneration-associated cognitive dysfunction.

## Molecular properties of klotho

### Proteolytic processing of klotho

**Figure 1:**
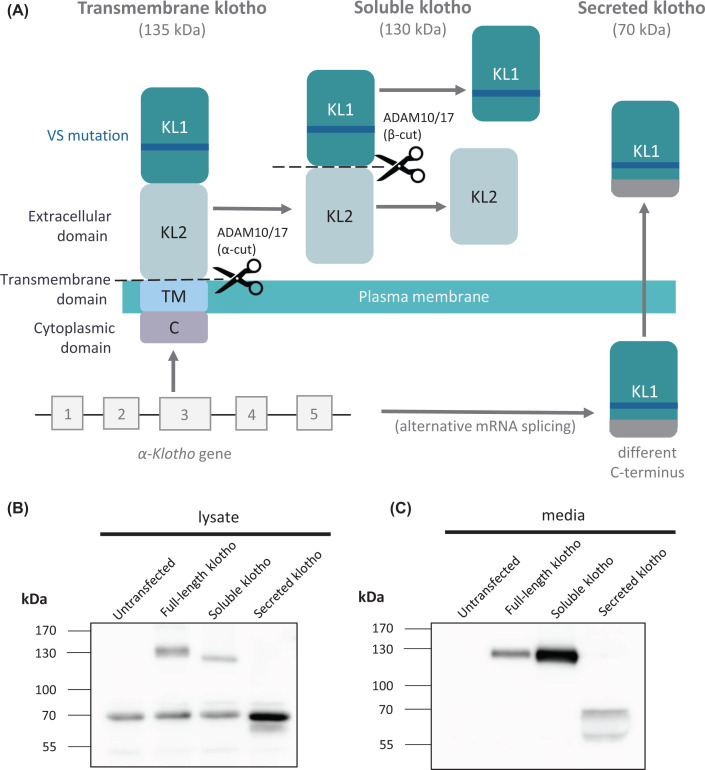
The different forms of α-klotho (**A**) The *α-klotho* gene produces a single pass type I transmembrane protein with a short intracellular domain (11 amino acids), a transmembrane domain (21 amino acids) and a large extracellular domain (980 amino acids). The extracellular domain contains two homologous repeats termed KL1 and KL2. The transmembrane form is shed from the membrane by the action of ADAM10/17 (α-cut), within the sequence LGSGTLGRF to produce soluble klotho. The second cleavage site (β-cut), also carried out by ADAM10/17, is between the KL1 and KL2 domains, within the sequence PPLPENQPL. The secreted form of klotho is produced by alternative splicing of the mRNA with a premature stop codon in exon 3, producing only the KL1 domain with an additional 15 amino acids at the C-terminus; with the sequence changing from DTTLSQFTDLNVYLW to SQLTKPISSLTKPYH. The secreted form is directly secreted from the cell. The klotho-VS polymorphism is located in the KL1 domain and is therefore present in all three forms of α-klotho. (**B**,**C**) HEK293 cells were stably transfected with the cDNAs encoding full-length transmembrane human klotho, soluble klotho or secreted klotho. Lysates (B) and media (C) were collected and immunoblotted with antibody LS-C500248. In the cell lysate full-length klotho migrates at 135 kDa, while in the media it migrates at 130 kDa due to its proteolytic shedding. The soluble and secreted forms of klotho migrate at 130 and 70 kDa, respectively, and are detected in both the cell lysate and media. In the media secreted klotho appears as two forms likely due to proteolytic processing.

The *α-klotho* gene is located on chromosome 13 and is translated into a single pass type 1 integral membrane protein. The klotho protein has a short intracellular domain (11 amino acids), a transmembrane domain (21 amino acids) and a large extracellular domain (980 amino acids; Figure 1A). The extracellular domain contains two repeat sequences of ∼440 amino acids each, termed the KL1 and KL2 domains. The 135-kDa transmembrane protein can be proteolytically cleaved in the juxtamembrane stalk region to produce a 130-kDa soluble, shed form of klotho (referred to here as soluble klotho but sometimes in the literature as shed klotho; Figure 1A,B). This cleavage in the juxtamembrane stalk, known as the α-cleavage, is carried out by a disintegrin and metalloproteinase domain-containing protein (ADAM) 10 and/or ADAM17 [10]. A second cleavage, known as the β-cleavage, occurs between the KL1 and KL2 domains and is likely carried out also by ADAM10 or ADAM17. The deletion of the α-cleavage site results in reduced α- and β-cleavage products, suggesting that the α-cleavage mainly occurs prior to the β-cleavage [11,12]. It is unclear whether the intact KL1 and KL2 domains in the transmembrane and soluble forms of klotho have different properties to the individual KL1 and KL2 domains produced following β-cleavage. However, it should be noted that the β-cleavage appears to be a minor event (Figure 1B,C) and in most cells and body fluids the 130-kDa form is the predominant form of soluble klotho. The transmembrane klotho is also cleaved by the β-site amyloid precursor protein (APP) cleaving enzyme 1 (BACE1) to generate a soluble form, and the transmembrane and cytosolic stub resulting from either ADAM or BACE1 cleavage is subject to intramembrane proteolysis by the presenilin-containing γ-secretase complex [13]. Such multistep proteolytic processing involving shedding of the ectodomain by an ADAM protease or BACE1 and then intramembrane proteolysis by the γ-secretase complex is a common feature of many cell surface transmembrane proteins, including APP and notch [14]. The *α-klotho* gene encodes another isoform derived through alternative mRNA splicing of exon 3; a secreted form of 70-kDa, which contains the KL1 sequence with an additional unique C-terminal sequence of 15 amino acids [15] (Figure 1A–C). The secreted and soluble forms of klotho are found in the cerebrospinal fluid (CSF), blood and urine [16,17]. As discussed below for klotho, a key issue with proteins that exist in multiple forms due to post-transcriptional processing, is to assign a particular function to a particular form.

### Does klotho have glycosidase activity?

The KL1 and KL2 domains have sequence similarity to glycosidases and have been reported to possess glycosidase activity, cleaving sialic acid from the carbohydrate chains attached to glycoproteins. For example, the glycosidase action of klotho on the calcium channels TRPC subfamily V member (TRPV) 5 and TRPV6 appears to allow their binding to galectin-1, leading to their clustering and retention on the plasma membrane, with a resultant increase in calcium channel activity [18,19]. However, the recent crystal structure of the extracellular domain of klotho revealed that both the KL1 and KL2 domains lack a key catalytic glutamate and have major conformational differences in the loops surrounding the catalytic pocket as compared with catalytically active glycosidases; differences that are incompatible with an intrinsic glycosidase activity [20]. Similar substitutions of key active site residues in β-klotho also indicate that this protein cannot function as an active glycosidase [21]. Thus, it is unlikely that klotho itself has glycosidase enzymatic activity but more likely that the KL domains bind sugars on glycoproteins or glycolipids promoting protein–protein or protein–lipid interactions, respectively.

## Klotho in the brain

In the brain, the highest level of klotho expression is in the choroid plexus, although klotho is also expressed in several other brain regions, including the hippocampus, cortex, cerebellum, striatum, substantia nigra, olfactory bulb and medulla [22,23]. Klotho is mainly expressed in both neurons and oligodendrocytes. Klotho expression in the brain starts *in utero* and continues to increase into adulthood [24,25]. However, klotho expression is reduced in the aged brain in monkeys, rats and mice [26] and in the CSF of humans [27]. The importance of klotho in healthy central nervous system (CNS) function was identified through klotho-deficient mice, where there were significantly fewer Purkinje cells in the cerebellum [1], diminished axonal transport [28] and cognitive impairment [29]. Klotho is also required for the proliferation and maturation of adult hippocampal neural progenitor cells [30], oligodendrocyte maturation and myelin integrity [31].

The choroid plexus contains epithelial cells with tight junctions and supports the CNS by producing CSF and growth factors, as well as providing a gateway for the entry of immune cells into the CNS (reviewed in [32]). By analogy with the kidney which produces soluble klotho for the blood circulation, the choroid plexus likely produces soluble and secreted klotho for the CSF [23]. Selective knockout of klotho in the choroid plexus of mice revealed the importance of choroid plexus-produced klotho [33]. Expression of the cytokine response factors, intracellular adhesion molecule 1 (ICAM1) and interferon regulatory factor 7 (IRF7) was increased in the choroid plexus of a Flox/Cre klotho knockout mouse model [33]. This suggests that klotho plays a regulatory role in the expression of inflammation-related genes. In the same model, decreased production of klotho in the choroid plexus caused enhanced macrophage infiltration into the CNS and promoted activation of microglia [33].

## Cell surface receptors for klotho

The atomic structure of a 1:1:1 ternary complex of the extracellular domain of klotho, the FGFR1c ligand-binding domain and FGF23 has been determined, revealing that klotho functions as an on-demand scaffold protein that promotes FGF23 signalling [20]. Most of the known roles of the FGF/klotho complex are in the renal tubules of the kidney where it aids phosphate regulation, vitamin D metabolism and the reabsorption of other ions [34]. The FGF/klotho complex also has a role in mediating cardiovascular homoeostasis via cardiomyocytes [35].

As the expression of klotho is limited to a few cell types, yet the protein affects the function of several non-klotho-expressing systems, it is likely that the soluble and secreted forms act as circulating hormones or ligands. However, the identity of the receptor(s) for the soluble and secreted forms of klotho remain unclear. Recently, klotho has been highlighted as a metabolic coupler between neurons and astrocytes [36]. Insulin acts upon neurons to stimulate the production and secretion of klotho which in turn stimulates astrocytic aerobic glycolysis and lactate release via FGF receptor 1 (FGFR1) and extracellular signal-regulated kinase 1/2 (Erk1/2) activation [36]. There is also a case for a potential klotho receptor on endothelial cells as they express FGFRs to which circulating klotho may bind to form a complex to activate signalling pathways [37]. However, there is little evidence that FGF23 is active in the brain (reviewed in [23]).

In HeLa cells and human embryonic kidney (HEK) cells, soluble klotho was reported to bind with a *K*_d_ of 3 µM to mono-sialogangliosides, which are highly enriched in the outer leaflet of cholesterol-rich lipid rafts [38]. Binding of soluble klotho to gangliosides modulated lipid raft organisation and inhibited lipid raft-dependent phosphoinositide 3-kinase (PI3K) signalling [38]. This implies that soluble klotho interacts with a raft-based protein or protein complex, possibly mediated by low-affinity interaction between the KL domains on klotho and mono-sialogangliosides on the membrane. However, in the brain, the cell surface receptors for klotho remain to be determined. Are there different receptors for the soluble and secreted forms of klotho? Are the receptors localised to one specific cell type in the brain? Unbiased screening approaches using the soluble and secreted forms of klotho as ligands will help answer these questions regarding the identity of the receptors for klotho in neurons and other cells of the CNS. Identificaton of the receptor(s) for klotho in the brain will aid in clarifying the potential molecular signalling pathways that the protein is involved in.

## Signalling pathways modulated by klotho

Various signalling pathways have been reported to be activated by klotho, including PI3K/Akt, Erk1/2, Ask1/p38 mitogen-activated protein kinase (MAPK), protein kinase R (PKR)-like endoplasmic reticulum kinase (PERK), those linked to the insulin and insulin-like growth factor (IGF)-1 receptors and Wnt1 [23,39,40]. For example, overexpression of the soluble form of klotho has been shown to suppress insulin/IGF-1 signalling in mice [2]. In the periphery, soluble klotho modulated PI3K/Akt signalling causing changes in calcium homoeostasis in cardiomyocytes [41] and decreased the abundance of transient receptor potential cation channels (TRPCs) 6 and TRPC3 on the surface of podocytes via inhibition of PI3K-dependent exocytosis [42]. Recently, soluble klotho has been shown to down-regulate Orai-mediated store-operated Ca^2+^ entry via PI3K-dependent signalling [43]. Klotho is also involved in regulation of cation channels such as Ca^2+^ and K^+^ [18]. For example, the purported sialidase activity of soluble klotho was observed to increase the abundance of K^+^ channels at the surface of non-neuronal cells via N-glycan modification [44].

In human neuroblastoma SH-SY5Y cells exposed to amyloid-β, recombinant soluble klotho treatment led to down-regulation of Wnt1, up-regulation of phosphorylated cyclic AMP response element binding protein (pCREB), and up-regulation of nuclear factor erythroid 2-related factor 2 (Nrf2) and heme oxygenase 1 (HO-1) expression [45]. Again in SH-SY5Y cells, expression of klotho blocked the thapsigargin-induced phosphorylation of the ER stress markers PERK and eukaryotic initiation factor 2α (eIF2α) [46]. Recombinant soluble klotho regulated several signalling proteins in rat oligodendrocytes *in vivo* and in a human oligodendrocytic cell line, including Wnt, nuclear factor κ-light-chain-enhancer of activated B cells (NFκB), p53, Akt and Erk [31,47].

In rat primary hippocampal neurons soluble klotho increased phosphorylation of PI3K/Akt and Erk [48]. In contrast, up-regulation of klotho, through expression of the transmembrane form, in the brain of senescence-accelerated prone (SAMP) 8 mice, an accelerated ageing model, decreased PI3K/Akt and Forkhead box class O1 (FoxO1) phosphorylation [49]. In transgenic mice, klotho overexpression significantly protected dopaminergic neurons against oxidative stress, in part by modulating the activation of Ask1/p38 MAPK [50]. From these limited published studies it is clear that there are still significant gaps in our understanding of the signalling pathways modulated by klotho in the brain.

In addition, several of the above studies are compounded by models in which *klotho* gene expression is increased which will increase the levels of transmembrane, soluble and secreted forms, thus making it difficult to distinguish which form of klotho is activating which signalling pathway (assuming that the various forms of klotho bind to separate receptors and/or modulate different signalling pathways). Studies either with recombinant forms of klotho or using viral vectors to selectively express a particular form of klotho, combined with single-cell RNA sequencing, will help to clarify the relative contribution of the different forms of klotho in modulating key signalling pathways in defined target cells in the brain.

## Effect of polymorphic variants of klotho on cognition

The most common klotho variant, KL-VS (which stands for klotho with valine and serine substitutions) consists of six single nucleotide polymorphisms (SNPs) that are always found together: three SNPs are in introns and do not alter splicing, the SNP at nucleotide 1155 causes no change in amino acid, while the other two SNPs result in the amino acid substitutions F352V and C370S, which are located in the KL1 domain and therefore occur in all forms of klotho (Figure 1A) [51–53]. In a transient transfection assay in HeLa cells, when incorporated into the secreted form of klotho, the F352V mutation on its own reduced by 6-fold the secretion of klotho, whereas the C370S mutation on its own increased by 2.9-fold the amount of klotho secreted [52]. The double mutation exhibited an intermediate phenotype (1.6-fold increase in secretion), providing an example of intragenic complementation in *cis* by human SNPs [52]. The KL-VS variant also increased klotho levels in sera of humans [3]. When incorporated into the transmembrane form of klotho, the F352V mutation on its own reduced proteolytic shedding in HEK293 cells, whereas the C370S mutation or the VS double mutation did not alter shedding of the protein compared with wildtype [51]. The F352V mutation led to a shorter half-life, but again this was attenuated in the VS variant [51]. When overexpressed the VS variant had more monomeric and less dimeric klotho, was a better binding partner for FGFR1, enhanced FGFR heterodimerisation and thus FGF23 signalling [51].

KL-VS homozygosity is associated with a reduced lifespan [52,54] and decreased cognitive function [55]. Whereas, heterozygosity for the KL-VS allele has been shown to protect against age-associated cognitive decline [55,56]. Furthermore, the KL-VS genetic variant of klotho was associated with enhanced cognition in three independent human cohorts and in a meta-analysis [3]. Such observations have prompted investigation into KL-VS allele status and the incidence of neurodegenerative disease. In individuals over 60 years, the KL-VS haplotype was associated with reduced risk of AD in the presence of apolipoprotein (Apo) Eε4 [57]. KL-VS heterozygosity in ApoEε4 individuals reduced the risk of progressing to mild cognitive impairment or AD, alongside increased amyloid-β levels in the CSF and reduced amyloid-β on positron emission topography scans [57]. The higher levels of amyloid-β in the CSF may be due to enhanced clearance from the brain. In ApoEε4 individuals with the KL-VS variant the amyloid-β burden did not exceed that of ApoEε4 negative individuals, suggesting heterozygosity of the VS haplotype may protect against ApoEε4-associated AD onset [58]. In a study of over 200 older adults, total tau and phosphorylated tau levels and cognitive deficits were reduced in KL-VS heterozygotes compared with non-carriers [59]. Heterozygosity of the KL-VS allele was correlated with a greater volume in the right dorsolateral prefrontal cortex (rDLPFC) and an enhanced executive function [55]. The rDLPFC is vulnerable to pathology and atrophy in AD [60]. Whereas KL-VS homozygosity was associated with a smaller rDLPFC volume and decreased executive function. Further investigation revealed that higher systemic klotho, via KL-VS heterozygosity, predicted greater connectivity between the rDLPFC to functional networks throughout the brain, including the anterior cingulate cortex and the right middle frontal gyrus [61]. In a separate study, individuals with KL-VS heterozygosity, relative to non-carriers, had slower cognitive decline and greater right frontal lobe volumes but also smaller white matter volumes and shorter survival [62]. Longitudinal cognitive trajectories indicated that KL-VS heterozygosity has an advantage in very late life, leading to the suggestion that the genotype-survival advantage of the KL-VS allele is age-dependent and mediated through differential cognition and brain volume [62]. Recently, no association of the KL-VS heterozygosity was found with cognition or brain structure in children and adolescents [56]. Other studies have assessed the KL-VS haplotype with cognitive ability in the same individuals from age 11 and again at age 79 [54]. From these various studies KL-VS heterozygosity appears to be protective in later life against age-related and neurodegeneration-associated cognitive decline, although the underlying molecular and cellular mechanisms by which this double mutation in klotho brings about these beneficial properties have yet to be understood.

The *klotho* SNP G395A is located in the promoter region and confers a higher affinity for transcription factors compared with wildtype, so was hypothesised to be a functional variant [63]. The G395A polymorphism is associated with reduced cognitive impairment in people over 90 years of age, as assessed by the mini-mental status examination (MMSE) [64]. The MMSE score indicated no difference in populations between 60 and 79 years with the G395A polymorphism, however, the intelligence quotient level was enhanced [65]. These data suggest that the G395A polymorphism also may be cognitively protective in older people only.

## Neuroprotective effects of klotho

**Figure 2:**
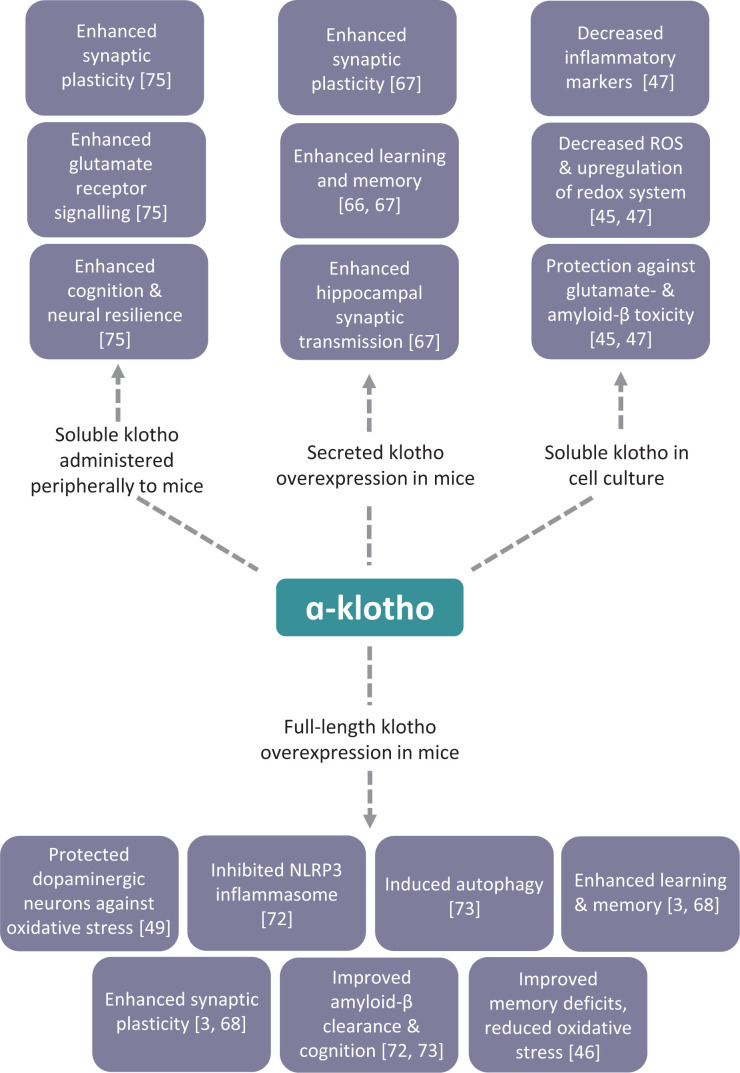
Reported neuroprotective effects of klotho in cell culture and mouse models Overview of the reported neuroprotective effects resulting from the overexpression of full-length or secreted klotho, or the administration of recombinant soluble klotho, in mouse models and administration of recombinant soluble klotho in cell culture models. See text for details including the uncertainty with several of the reported effects. Adapted from [23].

Despite the lack of information regarding the cell surface receptors and signalling pathways for klotho in the brain, studies with transgenic mice overexpressing the *klotho* gene have shed light on the neuroprotective properties of klotho and, when crossed with mouse models of neurodegenerative diseases, have highlighted the potential beneficial effect arising from enhancing klotho expression in such disorders (summarised in Figure 2). In transgenic mice that overexpress klotho throughout the body, the mice performed better in multiple tests of learning and memory than control mice [3]. Elevated klotho enhanced long-term potentiation, a form of synaptic plasticity widely studied as a cellular model for learning and memory. This effect of klotho to enhance cognition was via stimulus of the N-methyl-d-aspartate (NMDA) receptor subunit GluN2B [3]. Klotho-overexpressing mice had increased GluN2B synaptic expression in both the hippocampus and the frontal cortex [3]. Klotho elevation also increased expression of FOS, which is involved in memory consolidation and increased by NMDA receptor activation [3]. In wildtype mice, adenovirus expression of secreted klotho resulted in enhanced learning and memory 6 months after a single adenovirus injection into the CNS [66]. Viral expression of secreted klotho in the cortical area 1 (CA1) region of the hippocampus improved performance on the object recognition test and enhanced hippocampal synaptic transmission [67]. When klotho overexpressing transgenic mice were crossed with human APP transgenic mice, a model that displays AD-like pathology and behavioural deficits, the increased klotho levels ameliorated the cognitive deficits seen in the human APP transgenic mice, independently of amyloid-β accumulation [68]. In the klotho/human APP transgenic mice, GluN2B was enriched in post-synaptic densities and NMDA receptor-dependent synaptic plasticity in the hippocampus was enhanced [68].

Oxidative stress has long been implicated in ageing-related cognitive impairment in both old experimental animals and aged humans [69]. For example, oxidative damage to the synapse in the cerebral cortex and hippocampus during ageing contributes to the deficit of cognitive functions [70] and increased oxidative stress was associated with cognitive decline in a healthy population [71]. Oxidative stress contributed to the ageing-associated cognitive impairment in klotho mutant mice [29] and klotho knockout mice had a generalised increase in the global burden of oxidative stress in the CNS [2], indicating that klotho exerts antioxidant effects in the brain. Furthermore, lentivirus-mediated up-regulation of the transmembrane form of klotho improved ageing-related memory deficits and reduced oxidative stress in senescence-accelerated mice [49]. Rat primary hippocampal neurons treated with soluble klotho were protected against glutamate-induced and amyloid-β-induced oxidative damage, in part through regulation of the redox system via Akt-dependent induction of the thioredoxin/peroxiredoxin system [48]. Recently, recombinant soluble klotho was found to protect SH-SY5Y human neuroblastoma cells against amyloid-β toxicity through decreasing reactive oxygen species and increasing superoxide dismutase activity [45]. In addition, klotho reduced multiple inflammatory markers, NFκB, interleukin-1β and tumour necrosis factor-α (TNF-α), in cells exposed to amyloid-β [45].

In APP/PS1 mice intracerebral overexpression of full-length klotho cDNA by lentivirus injection ameliorated amyloid-β burden, neuronal and synaptic loss, and the cognitive deficits observed in this model of AD [72]. The klotho treatment significantly inhibited NACHT (neuronal apoptosis inhibitory protein, MHC class II transcription activator, incompatibility locus protein, and telomerase-associated protein), LRR (leucine-rich repeat) and PYD domain containing protein 3 (NLRP3) inflammasome and the subsequent transformation of microglia to the M2 type that may enhance microglia-mediated amyloid-β clearance [72]. In addition, klotho knockdown in primary human choroid plexus epithelial cells impaired their ability to transport amyloid-β [72]. Also, in APP/PS1 mice, intracerebroventricular injection of a lentiviral vector encoding klotho ameliorated the cognitive deficit and AD-like pathology in mice 3 months later [73]. Klotho-induced autophagy activation and protein kinase B/mammalian target of rapamycin inhibition, suggesting that up-regulation of klotho in the brain promotes the autophagic clearance of amyloid-β and protects against cognitive deficits [73]. From these various studies, klotho appears to convey neuroprotection against cognitive decline through multiple mechanisms: (i) promoting optimal synaptic function via activation of NMDA receptor signalling; (ii) stimulating the antioxidant defence system; (iii) reducing inflammation; (iv) promoting autophagy and (v) enhancing amyloid clearance. However, further work is required to validate many of these findings and to determine which are the key mechanisms responsible for the cognitive-enhancing effects of klotho *in vivo*.

## Approaches to increase klotho levels in the brain

Notwithstanding the limited knowledge of how klotho mediates neuroprotection and cognitive enhancement, the above observations (summarised in Figure 2) have led to the klotho pathway being considered as a potential therapeutic target for enhancing cognitive function [74]. As discussed above, overexpression of klotho using genetic approaches have provided convincing evidence that increasing klotho in the brain can enhance cognition and potentially reverse the cognitive-decline associated with ageing and AD. Several of these studies [3,49,68] have increased *klotho* expression throughout the body, so it is not clear whether the effects observed are due to increasing klotho in the CNS or the periphery. Targeted viral vector administration of klotho to discrete regions in the CNS has shown that klotho has direct beneficial actions on cells in the brain [66,67,73]. However, such genetic approaches in increasing klotho would be problematic in humans.

An alternative approach is to administer recombinant forms of klotho. This is exemplified in the study where soluble klotho administered peripherally induced cognitive enhancement and neural resilience in young, aged and transgenic α-synuclein mice [75]. This occurred through activation of the NMDA receptor subunit GluN2B with resultant enhancement of NMDA receptor-dependent synaptic plasticity [75]. Selective blockade of GluN2B subunits with the highly specific antagonist Ro 25-6981 abolished this acute effect of soluble klotho [75]. An intriguing aspect of this study was the ability of the peripherally administered klotho to cause an effect in the brain without seeming to cross the blood–brain barrier (BBB) [75]. This raises the possibility that the peripherally administered klotho may be acting in the cerebrovasculature, possibly directly on endothelial cells or other components of the neurovascular unit (pericytes, astrocytes), which then signal to the nearby neurons. Clearly, further work is required to validate the ability of peripherally administered klotho to activate NMDA receptors in the brain without crossing the BBB.

Another approach is to use small molecules to pharmacologically increase the expression of all forms of klotho or to selectively increase the soluble or secreted forms. In SAMP8 mice, the compound ligustilide elevated levels of klotho in the serum and choroid plexus, and reduced memory deficits and neuron loss [76]. Ligustilide inhibited the IGF1 pathway and induced FoxO1 activation, in addition to up-regulating klotho expression, in HEK293T cells [76]. Similarly, tetrahydroxystilbene glucoside was identified through studies on SAMP8 mice as increasing lifespan and increasing the level of neural klotho [77]. Using the *klotho* promoter to drive expression of luciferase, high-throughput screening was used to identify small molecules that promote *klotho* transcription [78]. FGF23 signalling assays and phosphorylation of Erk were assessed to determine that the increased klotho expression resulted in a functional change [78]. Whether any of the hits identified through this screen have progressed into *in vivo* studies has yet to be reported. In addition, it remains to be determined whether the gene-enhancing effects of ligustilide, tetrahydroxystilbene glucoside or other compounds identified through such genetic screens act solely via klotho or through activation of multiple genes.

**Figure 3:**
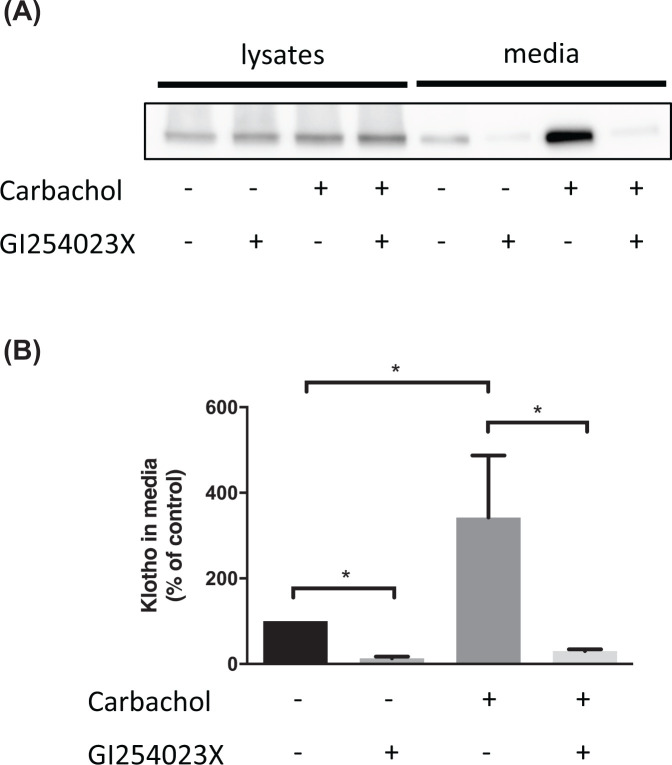
The muscarinic agonist carbachol promotes the shedding of klotho through activation of ADAM10 SH-SY5Y cells stably expressing klotho were incubated for 24 h with the muscarinic agonist carbachol (10 µM) and the selective ADAM10 inhibitor GI254023X (20 µM) as indicated. (**A**) Lysates and media were then prepared and immunoblotted for klotho with ab154163. (**B**) Quantitation of the amount of klotho in the media with the untreated cells set to 100%. Data are presented as mean ± SEM, *n*=4, Mann–Whitney test. * = *P*<0.05.

Pharmacologically promoting the proteolytic shedding of transmembrane proteins can have beneficial effects, for example, promoting the shedding of the prion protein through activation of ADAM10 with carbachol or acitretin reduces the binding and cytotoxicity of amyloid-β oligomers [79]. The proteolytic shedding of klotho can be stimulated with insulin [11] or the muscarinic agonist carbachol acting via activation of ADAM10 (Figure 3), indicating that it is feasible to increase the level of soluble klotho through pharmacologically enhancing ADAM10 and/or ADAM17 activity. As activation of ADAM10 also promotes the shedding of APP and increases the level of neuroprotective soluble APPα fragment [80], in addition to reducing the toxicity of amyloid-β oligomers through promoting the shedding of the prion protein [79], this approach would lead to neuroprotection through multiple routes. However, as both ADAM10 and ADAM17 have numerous other substrates, including some involved in tumourigenesis, activation of these proteases as a therapeutic approach has been questioned [81].

Finally, non-invasive and non-pharmacological approaches have been reported to increase klotho. A recent study showed blood klotho concentrations were increased after 2 weeks of moderate intensity training in men [82] and even after a single bout of high-intensity exercise [83,84]. The effect of diet on the expression of klotho has also been investigated. A low-calorie, high-protein diet significantly increased klotho expression in the brain of old rats and enhanced performance in the object recognition memory test [85]. However, such approaches as changes in exercise and diet will have multiple effects in the body, so linking a beneficial effect on cognition directly to alterations in klotho levels will be challenging.

## Concluding remarks

There is a growing body of evidence for klotho having cognitive-enhancing properties from genetic studies on the KL-VS variant to experiments directly increasing the level of klotho in the brain. However, several important questions on the mechanisms by which klotho acts remain unanswered. For example, which form of klotho (transmembrane, soluble or secreted) mediates its cognitive enhancing properties? Can the different forms substitute each other? What is the identity of the receptor(s) in the brain for the soluble and secreted forms of klotho and which signalling pathway(s) is activated by them in the brain to enhance cognition? What is the molecular basis for the beneficial effect of the heterozygous VS variant of klotho? Is it a gain of function, a loss of function or both? Given that KL-VS homozygosity appears to be detrimental, would too much klotho or overstimulation of its signalling pathways have toxic effects or be counterproductive to enhancing cognition? How does peripherally administered klotho mediate neuroprotection? Does it cross the BBB or act on non-neuronal cells in the neurovascular unit? Once more details are uncovered on the molecular and cellular mechanisms of action of the soluble and secreted forms of klotho in the brain, more targeted approaches to mimic the actions of klotho may be realised which will enable us to exploit its neuroprotective properties to tackle age- and neurodegeneration-associated cognitive dysfunction.

## Abbreviations

AD: Alzheimer’s disease
ADAM: a disintegrin and metalloproteinase
Apo: apolipoprotein
APP: amyloid precursor protein
BACE1: β-site amyloid precursor protein cleaving enzyme 1
BBB: blood–brain barrier
CNS: central nervous system
CSF: cerebrospinal fluid
Erk: extracellular signal-regulated kinase
FGF: fibroblast growth factor
FGFR: FGF receptor
FoxO1: forkhead box protein O1
HEK: human embryonic kidney
IGF: insulin-like growth factor
KL: klotho
KL-VS: klotho with valine and serine substitutions
MAPK: mitogen-activated protein kinase
MMSE: mini-mental status examination
NFκB: nuclear factor κ-light-chain-enhancer of activated B cells
NLRP3: NACHT-, LRR- and pyrin domain-containing protein 3
NMDA: N-methyl-d-aspartate
PERK: protein kinase R (PKR)-like endoplasmic reticulum kinase
PI3K: phosphoinositide 3-kinase
rDLPFC: right dorsolateral prefrontal cortex
SAMP: senescence-accelerated prone mouse
SNP: single nucleotide polymorphism

## References

[1] Kuro-o M., Matsumura Y., Aizawa H., Kawaguchi H., Suga T., Utsugi T. et al. (1997) Mutation of the mouse klotho gene leads to a syndrome resembling ageing. Nature 390, 45–51 10.1038/36285 9363890

[2] Kurosu H., Yamamoto M., Clark J.D., Pastor J.V., Nandi A., Gurnani P. et al. (2005) Suppression of aging in mice by the hormone Klotho. Science 309, 1829–1833 10.1126/science.1112766 16123266 PMC2536606

[3] Dubal D.B., Yokoyama J.S., Zhu L., Broestl L., Worden K., Wang D. et al. (2014) Life extension factor klotho enhances cognition. Cell Rep. 7, 1065–1076 10.1016/j.celrep.2014.03.076 24813892 PMC4176932

[4] Vo H.T., Laszczyk A.M. and King G.D. (2018) Klotho, the key to healthy brain aging? Brain Plast. 3, 183–194 10.3233/BPL-170057 30151342 PMC6091049

[5] Zou D., Wu W., He Y., Ma S. and Gao J. (2018) The role of klotho in chronic kidney disease. BMC Nephrol. 19, 285 10.1186/s12882-018-1094-z 30348110 PMC6198535

[6] Bian A., Neyra J.A., Zhan M. and Hu M.C. (2015) Klotho, stem cells, and aging. Clin. Interv. Aging 10, 1233–1243 26346243 10.2147/CIA.S84978 PMC4531025

[7] Hu M.C., Shiizaki K., Kuro-o M. and Moe O.W. (2013) Fibroblast growth factor 23 and Klotho: physiology and pathophysiology of an endocrine network of mineral metabolism. Annu. Rev. Physiol. 75, 503–533 10.1146/annurev-physiol-030212-183727 23398153 PMC3770142

[8] Yahata K., Mori K., Arai H., Koide S., Ogawa Y., Mukoyama M. et al. (2000) Molecular cloning and expression of a novel klotho-related protein. J. Mol. Med. 78, 389–394 10.1007/s001090000131 11043382

[9] Ito S., Kinoshita S., Shiraishi N., Nakagawa S., Sekine S., Fujimori T. et al. (2000) Molecular cloning and expression analyses of mouse betaklotho, which encodes a novel Klotho family protein. Mech. Dev. 98, 115–119 10.1016/S0925-4773(00)00439-1 11044614

[10] Chen C., Podvin S., Gillespie E., Leeman S.E. and Abraham C.R. (2007) Insulin stimulates the cleavage and release of the extracellular domain of Klotho by ADAM10 and ADAM17. Proc. Natl. Acad. Sci. U.S.A. 104, 19796–19801 10.1073/pnas.0709805104 18056631 PMC2148378

[11] Chen C., Tung T.Y., Liang J., Zeldich E., Tucker Zhou T.B., Turk B.E. et al. (2014) Identification of cleavage sites leading to the shed form of the anti-aging protein klotho. Biochemistry 53, 5579–5587 10.1021/bi500409n 25110992 PMC4151695

[12] Chen C., Li Y., Chen A.K., Rudy M.A., Nasse J.S., Zeldich E. et al. (2020) Identification of the cleavage sites leading to the shed forms of human and mouse anti-aging and cognition-enhancing protein Klotho. PLoS ONE 15, e0226382 10.1371/journal.pone.0226382 31929539 PMC6957300

[13] Bloch L., Sineshchekova O., Reichenback D., Reiss K., Saftig P., Kuro-o M. et al. (2009) Klotho is a substrate for α-, β- and γ-secretase. FEBS Lett. 583, 3221–3224 10.1016/j.febslet.2009.09.009 19737556 PMC2757472

[14] Lichtenthaler S.F., Lemberg M.K. and Fluhrer R. (2018) Proteolytic ectodomain shedding of membrane proteins in mammals—hardware, concepts, and recent developments. EMBO J. 37, e99456 10.15252/embj.201899456 29976761 PMC6068445

[15] Matsumura Y., Aizawa H., Shiraki-Iida T., Nagai R., Kuro-o M. and Nabeshima Y. (1998) Identification of the human klotho gene and its two transcripts encoding membrane and secreted klotho protein. Biochem. Biophys. Res. Commun. 242, 626–630 10.1006/bbrc.1997.8019 9464267

[16] Kunert S.K., Hartmann H., Haffner D. and Leifheit-Nestler M. (2017) Klotho and fibroblast growth factor 23 in cerebrospinal fluid in children. J. Bone Metab. 35, 215–226 10.1007/s00774-016-0746-y 27017221

[17] Imura A., Iwano A., Yohyama O., Tsuji Y., Nozaki K., Hashimoto N. et al. (2004) Secreted Klotho protein in sera and CSF: implication for post-translational cleavage in release of Klotho protein from cell membrane. FEBS Lett. 565, 143–147 10.1016/j.febslet.2004.03.090 15135068

[18] Chang Q., Hoefs S., van der Kemp A.W.T., Opala C.N., Bindels R.J. and Hoenderop J.G. (2005) The beta-glucuronidase klotho hydrolyzes and activates the TRPV5 channel. Science 310, 490–493 10.1126/science.1114245 16239475

[19] Lu P., Boros S., Chang Q., Bindels R.J. and Hoenderop J.G. (2008) The beta-glucuronidase klotho exclusively activates the epithelial Ca2+ channels TRPV5 and TRPV6. Nephrol. Dial. Transplant. 23, 3397–3402 10.1093/ndt/gfn291 18495742

[20] Chen G., Liu Y., Goetz R., Fu L., Jayaraman S., Hu M. et al. (2018) α-Klotho is a non-enzymatic molecular scaffold for FGF23 hormone signalling. Nature 553, 461–466 10.1038/nature25451 29342138 PMC6007875

[21] Lee S., Choi J., Mohanty J., Sousa L.P., Ome F., Pardon E. et al. (2018) Structures of β-klotho reveal a ‘zip code’-like mechanism for endocrine FGF signalling. Nature 553, 501–505 10.1038/nature25010 29342135 PMC6594174

[22] Abraham C.R., Chen C., Cuny G.D., Glicksman M.A. and Zeldich E. (2012) Small-molecule Klotho enhancers as novel treatment of neurodegeneration. Future Med. Chem. 4, 1671–1679 10.4155/fmc.12.134 22924505 PMC3564652

[23] Cararo-Lopes M.M., Mazucanti C.H., Scavone C., Kawamoto E.M. and Berwick D.C. (2017) The relevance of α-KLOTHO to the central nervous system: some key questions. Ageing Res. Rev. 36, 137–148 10.1016/j.arr.2017.03.003 28323064

[24] Takeshita K., Fujimori T., Kurotaki Y., Honjo H., Tsujikawa H., Yasui K. et al. (2004) Sinoatrial node dysfunction and early unexpected death of mice with a defect of klotho gene expression. Circulation 109, 1776–1782 10.1161/01.CIR.0000124224.48962.32 15037532

[25] Clinton S.M., Glover M.E., Maltare A., Laszczyk A.M., Mehi S.J., Simmons R.K. et al. (2013) Expression of klotho mRNA and protein in rat brain parenchyma from early postnatal development into adulthood. Brain Res. 1527, 1–14 10.1016/j.brainres.2013.06.044 23838326 PMC3756829

[26] Duce J.A., Podvin S., Hollander W., Kipling D., Rosene D.L. and Abraham C.R. (2008) Gene profile analysis implicates Klotho as an important contributor to aging changes in brain white matter of the rhesus monkey. Glia 56, 106–117 10.1002/glia.20593 17963266

[27] Semba R.D., Moghekar A.R., Hu J., Sun K., Turner R., Ferrucci L. et al. (2014) Klotho in the cerebrospinal fluid of adults with and without Alzheimer’s disease. Neurosci. Lett. 558, 37–40 10.1016/j.neulet.2013.10.058 24211693 PMC4037850

[28] Uchida A., Komiya Y., Tashiro T., Yorifuji H., Kishimoto T., Nabeshima Y. et al. (2001) Neurofilaments of Klotho, the mutant mouse prematurely displaying symptoms resembling human aging. J. Neurosci. Res. 64, 364–370 10.1002/jnr.1087 11340643

[29] Nagai T., Yamada K., Kim H., Kim Y., Noda Y., Imura A. et al. (2003) Cognition impairment in the genetic model of aging klotho gene mutant mice: a role of oxidative stress. FASEB J. 17, 50–52 10.1096/fj.02-0448fje 12475907

[30] Laszczyk A.M., Fox-Quick S., Vo H.T., Nettles D., Pugh P.C., Overstreet-Wadiche L. et al. (2017) Klotho regulates postnatal neurogenesis and protects against age-related spatial memory loss. Neurobiol. Aging 59, 41–54 10.1016/j.neurobiolaging.2017.07.008 28837861 PMC5612914

[31] Chen C., Slaone J.A., Li H., Aytan N., Giannaris E.L., Zeldich E. et al. (2013) The antiaging protein klotho enhances oligodendrocyte maturation and myelination of the CNS. J. Neurosci. 33, 1927–1939 10.1523/JNEUROSCI.2080-12.2013 23365232 PMC3711388

[32] Kaur C., Rathnasamy G. and Ling E. (2016) The choroid plexus in healthy and diseased brain. J. Neuropathol. Exp. 75, 198–213 10.1093/jnen/nlv030 26888305

[33] Zhu L., Stein L.R., Kim D., Ho K., Yu G., Zhan L. et al. (2018) Klotho controls the brain–immune system interface in the choroid plexus. Proc. Natl. Acad. Sci. U.S.A. 115, e11388–e11396 10.1073/pnas.1808609115 30413620 PMC6275534

[34] Haussler M.R., Whitfield G.K., Kaneko I., Forster R., Saini R., Hsieh J. et al. (2012) The role of vitamin D in the FGF23, klotho, and phosphate bone-kidney endocrine axis. Rev. Endocr. Metab. Disord. 13, 57–69 10.1007/s11154-011-9199-8 21932165 PMC3288475

[35] Pi M., Ye R., Han X., Armstrong B., Liu X., Chen Y. et al. (2018) Cardiovascular interactions between fibroblast growth factor-23 and angiotensin II. Sci. Rep. 8, 12398 10.1038/s41598-018-30098-1 30120363 PMC6098163

[36] Mazucanti C.H., Kawamoto E.M., Mattson M.P., Scavone C. and Camandola S. (2019) Activity-dependent neuronal Klotho enhances astrocytic aerobic glycolysis. J. Cereb. Blood Flow Metab. 39, 1544–1556 10.1177/0271678X18762700 29493420 PMC6681535

[37] Chung C., Chang Y., Ding Y., Lim K., Liu Q., Zhu L. et al. (2017) α-Klotho expression determines nitric oxide synthesis in response to FGF-23 in human aortic endothelial cells. PLoS ONE 12, e0176817 10.1371/journal.pone.0176817 28463984 PMC5413063

[38] Dalton G., An S., Al-Juboori S.I., Nischan N., Yoon J., Dobrinskikh E. et al. (2017) Soluble klotho binds monosialoganglioside to regulate membrane microdomains and growth factor signaling. Proc. Natl. Acad. Sci. U.S.A. 114, 752–757 10.1073/pnas.1620301114 28069944 PMC5278494

[39] Sopjani M., Rinnerthaler M., Kruja J. and Dermaku-Sopjani M. (2015) Intracellular signaling of the aging suppressor protein Klotho. Curr. Mol. Med. 15, 27–37 10.2174/1566524015666150114111258 25601466

[40] Dërmaku-Sopjani M., Kolgeci S., Abazi S. and Sopjani M. (2013) Significance of the anti-aging protein Klotho. Mol. Membr. Biol. 30, 369–385 10.3109/09687688.2013.837518 24124751

[41] Hung Y., Chen Y., Huang S., Lu Y., Lin Y., Kao Y. et al. (2020) Klotho modulates electrical activity and calcium homeostasis in pulmonary vein cardiomyocytes via PI3K/Akt signalling. EP Europace 22, 1132–1141 10.1093/europace/euaa100 32627831

[42] Kim J.H., Xie J., Hwang K.H., Wu Y.L., Oliver N., Eom M. et al. (2017) Klotho may ameliorate proteinuria by targeting TRPC6 channels in podocytes. Clin. J. Am. Soc. Nephrol. 28, 140–151 10.1681/ASN.2015080888 PMC5198269 27151926

[43] Kim J., Park E.Y., Hwang K., Park K., Choi S.J. and Cha S. (2021) Soluble αKlotho downregulates Orai1-mediated store-operated Ca2+ entry via PI3K-dependent signaling. Eur. J. Physiol. 473, 647–658 10.1007/s00424-020-02510-1 PMC8049930 33386992

[44] Huang C. (2012) Regulation of ion channels by secreted klotho. In: Kuro-o M. (eds) Endocrine FGFs and Klothos. Adv. Exp. Med. 728, Springer, New York, NY. 10.1007/978-1-4614-0887-1_7 22396165

[45] Sedighi M., Baluchnejadmojarad T., Afshin-Majd S., Amiri M., Aminzade M. and Roghani M. (2021) Anti-aging klotho protects SH-SY5Y cells against amyloid β1-42 neurotoxicity: involvement of Wnt1/pCREB/Nrf2/HO-1 signaling. J. Mol. Neurosci. 71, 19–27 10.1007/s12031-020-01621-9 32627121

[46] Banerjee S., Zhao Y., Sarkar P.S., Rosenblatt K.P., Tilton R.G. and Choudhary S. (2013) Klotho ameliorates chemically induced endoplasmic reticulum (ER) stress signaling. Cell. Physiol. Biochem. 31, 659–672 10.1159/000350085 23711492

[47] Chen C., Li H., Liang J., Hixson K., Zeldich E. and Abraham C.R. (2015) The anti-aging and tumor suppressor protein Klotho enhances differentiation of a human oligodendrocytic hybrid cell line. J. Mol. Neurosci. 55, 79–90 10.1007/s12031-014-0336-1 PMC5154549 24907942

[48] Zeldich E., Chen C., Colvin T.A., Bove-Fenderson E.A., Liang J., Tucker Zhou T.B. et al. (2014) The neuroprotective effect of Klotho is mediated via regulation of members of the redox system. J. Biol. Chem. 289, 24700–24715 10.1074/jbc.M114.567321 25037225 PMC4148892

[49] Zhou H., Zeng C., Yang T., Long F., Kuang X. and Du J. (2018) Lentivirus-mediated klotho up-regulation improves aging-related memory deficits and oxidative stress in senescence-accelerated mouse prone-8 mice. Life Sci. 200, 56–62 10.1016/j.lfs.2018.03.027 29544758

[50] Brobey R.K., German D., Sonsalla P.K., Gurnani P., Pastor J., Hsieh C. et al. (2015) Klotho protects dopaminergic neuron oxidant-induced degeneration by modulating ASK1 and p38 MAPK signaling pathways. PLoS ONE 10, e0139914 10.1371/journal.pone.0139914 26452228 PMC4599800

[51] Tucker Zhou T.B., King G.D., Chen C. and Abraham C.R. (2013) Biochemical and functional characterization of the klotho-VS polymorphism implicated in aging and disease risk. J. Biol. Chem. 288, 36302–36311 10.1074/jbc.M113.490052 24217253 PMC3868745

[52] Arking D.E., Krebsova A., Macek M. Sr, Macek M. Jr, Arking A., Mian I.S. et al. (2002) Association of human aging with a functional variant of klotho. Proc. Natl. Acad. Sci. U.S.A. 99, 856–861 10.1073/pnas.022484299 11792841 PMC117395

[53] Mengel-From J., Soerensen M., Nygaard M., McGue M., Christensen K. and Christiansen L. (2016) Genetic variants in KLOTHO associate with cognitive function in the oldest old group. J. Gerontol. 71, 1151–1159 10.1093/gerona/glv163 PMC4978356 26405063

[54] Deary I.J., Harris S.E., Fox H.C., Hayward C., Wright A.F., Starr J.M. et al. (2005) KLOTHO genotype and cognitive ability in childhood and old age in the same individuals. Neurosci. Lett. 378, 22–27 10.1016/j.neulet.2004.12.005 15763166

[55] Yokoyama J.S., Sturm V.E., Bonham L.W., Klein E., Arfanakis K., Yu L. et al. (2015) Variation in longevity gene KLOTHO is associated with greater cortical volumes. Ann. Clin. Transl. Neurol. 2, 215–230 10.1002/acn3.161 25815349 PMC4369272

[56] de Vries C.F., Staff R.T., Noble K.G., Muetzel R.L., Vernooij M.W., White T. et al. (2020) Klotho gene polymorphism, brain structure and cognition in early-life development. Brain Imaging Behav. 14, 213–225 10.1007/s11682-018-9990-1 30393836 PMC6588504

[57] Belloy M.E., Napolioni V., Han S.S., Guen Y.L. and Greicius M.D. (2020) Association of klotho-VS heterozygosity with risk of Alzheimer disease in individuals who carry APOE4. JAMA Neurol. 77, 849–862 10.1001/jamaneurol.2020.0414 32282020 PMC7154955

[58] Erickson C.M., Schultz S.A., Oh J.M., Darst B.F., Ma Y., Norton D. et al. (2019) KLOTHO heterozygosity attenuates APOE4-related amyloid burden in preclinical AD. Neurology 92, e1878–e1889 10.1212/WNL.0000000000007323 30867273 PMC6550504

[59] Driscoll I., Ma Y., Gallagher C.L., Johnson S.C., Asthana S., Hermann B.P. et al. (2021) Age-related tau burden and cognitive deficits are attenuated in KLOTHO KL-VS heterozygotes. J. Alzheimers Dis. 79, 1297–1305 10.3233/JAD-200944 33427737 PMC9472657

[60] Lunnon K., Smith R., Hannon E., De Jager P., Srivastava G., Volta M. et al. (2014) Cross-tissue methylomic profiling strongly implicates a role for cortex-specific deregulation of ANK1 in Alzheimer’s disease neuropathology. Nat. Neurosci. 17, 1164–1170 10.1038/nn.3782 25129077 PMC4410018

[61] Yokoyama J.S., Marx G., Brown J.A., Bonham L.W., Wang D., Coppola G. et al. (2017) Systemic klotho is associated with KLOTHO variation and predicts intrinsic cortical connectivity in healthy human aging. Brain Imaging Behav. 11, 391–400 10.1007/s11682-016-9598-2 27714549 PMC5382127

[62] de Vries C.F., Staff R.T., Harris S.E., Chapko D., Williams D.S., Reichert P. et al. (2017) Klotho, APOEε4, cognitive ability, brain size, atrophy, and survival: a study in the Aberdeen Birth Cohort of 1936. Neurobiol. Aging 55, 91–98 10.1016/j.neurobiolaging.2017.02.019 28431289

[63] Kawano K., Ogata N., Chiano M., Molloy H., Kleyn P., Spector T.D. et al. (2002) Klotho gene polymorphisms associated with bone density of aged postmenopausal women. Bone Miner. Res. 17, 1744–1751 10.1359/jbmr.2002.17.10.1744 12369777

[64] Hao Q., Ding X., Gao L., Yang M. and Dong B. (2016) G-395A polymorphism in the promoter region of the KLOTHO gene associates with reduced cognitive impairment among the oldest old. Age 38, 7 10.1007/s11357-015-9869-7 26732817 PMC5005865

[65] Shimokata H., Ando F., Fukukawa Y. and Nishita Y. (2006) Klotho gene promoter polymorphism and cognitive impairment. Geriatr. Gerontol. Int. 6, 136–141 10.1111/j.1447-0594.2006.00335.x

[66] Massó A., Sánchez A., Bosch A., Giménez-Llort L. and Chillón M. (2017) Secreted αKlotho isoform protects against age-dependent memory deficits. Mol. Psychiatry 23, 1937–1947 10.1038/mp.2017.211 29086766

[67] Li D., Jing D., Liu Z., Chen Y., Huang F. and Behnisch T. (2019) Enhanced expression of secreted α-klotho in the hippocampus alters nesting behaviour and memory formation in mice. Front. Cell Neurosci. 13, 133 10.3389/fncel.2019.00133 31001090 PMC6454015

[68] Dubal D.B., Zhu L., Sanchez P.E., Worden K., Broestl L., Johnson E. et al. (2015) Life extension factor klotho prevents mortality and enhances cognition in hAPP transgenic mice. J. Neurosci. 35, 2358–2371 10.1523/JNEUROSCI.5791-12.2015 25673831 PMC4323521

[69] Harman D. (1981) The aging process. Proc. Natl. Acad. Sci. U.S.A. 78, 7124–7128 10.1073/pnas.78.11.7124 6947277 PMC349208

[70] Fukui K., Omoi N., Hayasaka T., Shinnkai T., Suzuki S., Abe K. et al. (2002) Cognitive impairment of rats caused by oxidative stress and aging, and its prevention by vitamin E. N.Y. Acad. Sci. 959, 275–284 10.1111/j.1749-6632.2002.tb02099.x 11976202

[71] Hajjar I., Hayek S.S., Goldstein F.C., Martin G., Jones D.P. and Quyyumi A. (2018) Oxidative stress predicts cognitive decline with aging in healthy adults: an observational study. J. Neuroinflammation 15, 17 10.1186/s12974-017-1026-z 29338747 PMC5771063

[72] Zhao Y., Zeng C., Li X., Yang T., Kuang X. and Du J. (2020) Klotho overexpression improves amyloid‐β clearance and cognition in the APP/PS1 mouse model of Alzheimer’s disease. Aging Cell 19, e13239 10.1111/acel.13239 PMC7576297 32964663

[73] Zeng C., Yang T., Zhou H., Zhao Y., Kuang X., Duan W. et al. (2019) Lentiviral vector-mediated overexpression of Klotho in the brain improves Alzheimer's disease-like pathology and cognitive deficits in mice. Neurobiol. Aging 78, 18–28 10.1016/j.neurobiolaging.2019.02.003 30851437

[74] Abraham C.R., Mullen P.C., Tucker-Zhou T., Chen C.D. and Zeldich E. (2016) Klotho is a neuroprotective and cognition-enhancing protein. Vitam. Horm. 101, 215–238 10.1016/bs.vh.2016.02.004 27125744

[75] Leon J., Moreno A.J., Garay B.I., Chalkley R.J., Burlingame A.L., Wang D. et al. (2017) Peripheral elevation of a Klotho fragment enhances brain function and resilience in young, aging, and α-synuclein transgenic mice. Cell Rep. 20, 1360–1371 10.1016/j.celrep.2017.07.024 28793260 PMC5816951

[76] Kuang X., Chen Y., Wang L., Li Y., Liu K., Zhang M. et al. (2014) Klotho upregulation contributes to the neuroprotection of ligustilide in an Alzheimer’s disease mouse model. Neurobiol. Aging 35, 169–178 10.1016/j.neurobiolaging.2013.07.019 23973442

[77] Zhou L., Mo H., Miao J., Zhou D., Tan R.J., Hou F.F. et al. (2015) Klotho ameliorates kidney injury and fibrosis and normalizes blood pressure by targeting the renin-angiotensin system. Am. J. Pathol. 185, 3211–3223 10.1016/j.ajpath.2015.08.004 26475416 PMC4729238

[78] King G.D., Chen C., Huang M.M., Zeldich E., Brazee P.L., Schuman E.R. et al. (2012) Identification of novel small molecules that elevate Klotho expression. Biochem. J. 441, 453–461 10.1042/BJ20101909 21939436 PMC3677209

[79] Jarosz-Griffiths H.H., Corbett N.J., Rowland H.A., Fisher K., Jones A.C., Baron J. et al. (2019) Proteolytic shedding of the prion protein via activation of metallopeptidase ADAM10 reduces cellular binding and toxicity of amyloid-β oligomers. J. Biol. Chem. 294, 7085–7097 10.1074/jbc.RA118.005364 30872401 PMC6497954

[80] Andrew R.J., Kellett K.A.B., Thinakaran G. and Hooper N.M. (2016) A Greek tragedy: the growing complexity of Alzheimer amyloid precursor protein proteolysis. J. Biol. Chem. 291, 19235–19244 10.1074/jbc.R116.746032 27474742 PMC5016663

[81] Mockett B.G., Richter M., Abraham W.C. and Müller U.C. (2017) Therapeutic potential of secreted amyloid precursor protein APPsα. Front. Mol. Neurosci. 10, 30 10.3389/fnmol.2017.00030 28223920 PMC5293819

[82] Middelbeek R.J., Motiani P., Brandt N., Nigro P., Zheng J., Virtanen K.A. et al. (2021) Exercise intensity regulates cytokine and klotho responses in men. Nutr. Diabetes 11, 5 10.1038/s41387-020-00144-x 33414377 PMC7791135

[83] Tan S., Chu M.M., Toussaint N.D., Cai M.M., Hewitson T.D. and Holt S.G. (2018) High-intensity physical exercise increases serum α-klotho levels in healthy volunteers. J. Circ. Biomark 7, 1849454418794582 10.1177/1849454418794582 30147756 PMC6100126

[84] Santos-Dias A., MacKenzie B., Oliveira-Junior M.C., Moyses R.M., Consolim-Colombo F.M. and Vieira R.P. (2017) Longevity protein klotho is induced by a single bout of exercise. Br. J. Sports Med. 51, 549–550 10.1136/bjsports-2016-096139 27251899

[85] Shafie A., Rahimi A.M., Ahmadi I., Nabavizadeh F., Ranjbaran M. and Ashabi G. (2020) High-protein and low-calorie diets improved the anti-aging Klotho protein in the rats’ brain: the toxic role of high-fat diet. Nutr. Metab. 17, 86 10.1186/s12986-020-00508-1 PMC7559193 33072166

